# Severe Mental Health Screening Signals Among Multinational Hajj Pilgrims in 2025: Implications for Multilingual Healthcare Encounters and Referral Planning

**DOI:** 10.3390/healthcare14152276

**Published:** 2026-07-25

**Authors:** Majed Alqahtani, Mohammed AlGabgab, Moharmis M. Alolyani, Naif Alqurashi, Osama A. Samarkandi

**Affiliations:** 1 Basic Sciences Department, Prince Sultan College for Emergency Medical Services, King Saud University, Riyadh 11466, Saudi Arabia; maalqahtani@ksu.edu.sa (M.A.); malgabgab@ksu.edu.sa (M.A.); 2 Emergency Medical Services Department, Prince Sultan College for Emergency Medical Services, King Saud University, Riyadh 11466, Saudi Arabia; malolyani@ksu.edu.sa; 3 Department of Accidents and Trauma, Prince Sultan bin Abdulaziz College for Emergency Medical Services, King Saud University, Riyadh 11466, Saudi Arabia; nalqurashi1@ksu.edu.sa

**Keywords:** Hajj, mass gatherings, healthcare encounters, mental health screening, suicidal ideation, psychotic-like experiences, mood instability, multilingual assessment

## Abstract

**Background/Objectives**: Large multinational mass gatherings can create healthcare encounters in which severe mental health screening signals require rapid recognition, confidential assessment, language support, and referral. During Hajj, culturally and linguistically diverse pilgrims may first disclose severe psychological symptoms in general medical or field settings rather than through specialist psychiatric pathways. This study estimated the prevalence, overlap, and adjusted correlates of severe screen-positive mental health symptom signals among adult multinational Hajj pilgrims in 2025 and considered implications for multilingual healthcare encounters and referral planning. **Methods**: This cross-sectional field screening study analyzed de-identified coded data from 2171 adult pilgrims aged 18–95 years. Participants completed a structured, interview-administered multilingual mental health screening survey comprising 23 binary symptom items. The analysis focused on suicide-related symptoms, psychotic-like symptoms, and mood-instability/bipolar-spectrum symptoms. A severe symptom cluster was defined as positivity in at least one of these domains. Prevalence estimates with Wilson 95% confidence intervals, domain overlap, and multivariable logistic regression models were used. **Results**: Severe symptom cluster positivity was identified in 428 participants (19.7%; 95% CI 18.1–21.4). Mood-instability/bipolar-spectrum signals were most frequent (13.3%; 95% CI 11.9–14.8), followed by psychotic-like symptoms (8.0%; 95% CI 6.9–9.2) and suicide-related symptoms (5.6%; 95% CI 4.7–6.6). Among cluster-positive participants, 297 endorsed one severe domain, 108 endorsed two domains, and 23 endorsed all three. Current physical illness was independently associated with all severe outcomes, whereas prior psychiatric history was not independently associated with suicide-related symptoms. **Conclusions**: Severe screen-positive mental health signals were present in a clinically relevant minority of pilgrims. The findings support integrating confidential severe symptom screening, interpreter-supported assessment, psychological first aid, and clear referral pathways into general Hajj healthcare encounters, while emphasizing that screen-positive findings are not psychiatric diagnoses.

## 1. Introduction

Hajj is a recurrent multinational mass gathering that brings together pilgrims from diverse countries, languages, cultural backgrounds, health-system traditions, and levels of access to mental healthcare. Health planning for Hajj has traditionally emphasized infectious disease prevention, heat exposure, chronic disease, injuries, emergency preparedness, and health-service capacity [1,2,3,4,5]. These priorities remain central, but they do not fully address the psychological needs that may emerge during travel, crowding, sleep disruption, physical strain, and interaction with unfamiliar health systems. Recent Hajj 2025 healthcare research has also shown that simple field documentation flags can support care-pathway planning in mass-gathering settings, provided they are interpreted as operational prompts rather than standalone prediction tools [6].

For many pilgrims, mental health needs may first become visible in general medical, emergency, campaign, or field encounters rather than through specialist psychiatric services. Disclosure may be affected by stigma, privacy concerns, language barriers, cultural idioms of distress, fear of consequences, or uncertainty about available services [7,8]. In this setting, the ability of healthcare teams to recognize severe screening signals and connect pilgrims to support is a healthcare-delivery issue rather than a purely psychiatric diagnostic problem.

Severe symptom signals such as suicide-related thoughts, psychotic-like experiences, or marked mood instability may require confidential assessment, psychological first aid, culturally sensitive communication, safety review, and referral planning [9,10,11]. At the same time, brief field screening cannot establish psychiatric diagnoses. Screening identifies signals that may warrant further assessment; it cannot diagnose suicidal behavior, psychotic disorder, bipolar disorder, or any other psychiatric condition [12]. This distinction is especially important in multilingual pilgrimage settings, where literal item endorsement may be shaped by language, spirituality, fatigue, distress, or social context.

Empirical psychiatric evidence from Hajj remains limited. A diagnostic interview study among 513 pilgrims reported at least one psychiatric disorder in 7.2% and suicide risk in 6.2% using the Mini International Neuropsychiatric Interview [13]. Referral-based evidence has also described psychiatric morbidity among pilgrims who reached specialist hospital services [14]. These studies are informative, but they do not directly address large-scale multilingual field screening for crisis-relevant symptom signals among multinational pilgrims in general healthcare-associated contexts.

The present study estimated the prevalence, item-level endorsement, overlap, and adjusted correlates of severe screen-positive mental health symptom signals among adult multinational Hajj pilgrims during the 2025 season. The objective was to generate healthcare-relevant evidence for confidential multilingual screening, clinical vigilance, and referral planning in mass-gathering settings while explicitly avoiding diagnostic overinterpretation.

## 2. Materials and Methods

### 2.1. Study Design, Setting, and Participants

This was a focused analysis of an original cross-sectional field screening dataset collected during the 2025 Hajj season. The parent study assessed mental health symptoms among multinational pilgrims using a structured, interview-administered multilingual screening survey. Interviews were conducted after pilgrims arrived at accommodation sites by trained psychology personnel, with language support when required.

Eligible participants were adult Hajj pilgrims aged 18 years or older with coded responses available for the severe symptom domains of interest. The initial field dataset included 2174 pilgrims. Three participants younger than 18 years were excluded, yielding a final analytic sample of 2171 adults aged 18–95 years. The present analysis used de-identified coded data and did not involve additional participant recruitment beyond the original field screening.

For clarity, the parent study refers to the broader original Hajj 2025 mental health field screening project from which this focused severe symptom analysis was derived; it was not a separately published parent dataset at the time of this submission. Participant characteristics presented in Table 1 were extracted directly from the same de-identified parent field screening dataset used for the present analysis.

Sampling followed a pragmatic field-based convenience approach within accessible Hajj mission accommodation sites. Participants were approached during arranged access windows after arrival at accommodation sites, subject to mission-office coordination, field-team access, crowding, and participant willingness to take part. The sample was therefore not probability-based and should not be interpreted as representative of all Hajj pilgrims; this sampling feature was considered when interpreting prevalence estimates and potential selection bias.

### 2.2. Screening Instrument, Multilingual Administration, and Field Procedures

Data were collected using the Mental Illness Brief Screening Survey, a 23-item binary screening instrument designed for brief interview administration in Hajj field settings. Responses were coded as 0 = no and 1 = yes. The broader instrument covered depressive symptoms, anxiety symptoms, suicide-related symptoms, psychotic-like symptoms, adjustment-related symptoms, and mood-related/bipolar-spectrum symptoms. The instrument was used for preliminary symptom screening and early recognition of participants who might require further assessment or referral; it was not designed or used as a diagnostic interview.

Completed field responses were organized in Microsoft Excel (Microsoft Corporation, Redmond, WA, USA) and transformed into an analysis-ready coded dataset. The 23 screening items retained their binary 0/1 coding, and demographic, clinical history, physical illness, and language-support variables were decoded from the same parent field dataset using predefined categories. Derived severe-domain variables were then created from the relevant item groups after exclusion of ineligible records. Table 1 therefore describes the analytic sample derived from the parent field screening dataset rather than a separate external dataset.

The survey was administered through direct individual interviews after pilgrims arrived at their accommodation sites. Interviews were conducted in quiet or semi-private settings when feasible and typically required approximately 3–5 min. Field data collection was conducted by 10 trained psychology personnel, including two master-level psychologists and eight bachelor-level psychologists. Field coordinators liaised with Hajj mission offices to arrange interview timing, access, and team movement routes, particularly during peak crowding periods.

The parent study used 13 language-specific survey versions and interpreter-supported administration according to pilgrims’ communication needs. Twelve language-support personnel assisted during interviews and were distinct from the survey-tool reviewers. Table 1 reports the three most frequent administration/support streams and a combined category for the remaining language-specific or interpreter-supported streams. For Hindi-speaking participants, Urdu and/or English-assisted administration was used when applicable because the field survey form was implemented as an Urdu language version rather than a separate Hindi language form.

Before field implementation, the survey tool underwent field-applicability and multilingual clarity review between May and July 2025. Twelve international multilingual reviewers from different national and language backgrounds, many working with international Hajj missions, reviewed the survey tool only. The review used a mixed written feedback and discussion format, including structured written feedback and face-to-face review meetings. The reviewer group included three emergency care clinicians, two Hajj health-system planners, two mental health professionals, two psychology PhD holders, and three campaign or medical mission physicians. One psychiatrist provided clinical review input. Feedback was organized in Excel and used to refine screening triggers, response levels, referral language, stakeholder roles, and interpreter-supported handover wording.

Field procedures included safeguards for sensitive disclosures. Interviewers were instructed not to pressure participants who declined to answer, to use simplified language or interpreter support when communication barriers occurred, and to stop the interview if severe distress or suicide-related indicators emerged. In such cases, the responsible coordinator was notified and psychological support or referral pathways were activated. Interviewers did not provide field diagnosis or treatment advice.

### 2.3. Outcome Definitions and Covariates

Three severe screen-positive domains were selected a priori because of their relevance to confidential assessment, safety review, and referral planning. The suicide-related domain was defined as endorsement of at least one of Q11–Q13: thoughts about death or wishing to die during the past month, thoughts of self-harm or suicide even briefly, or any previous suicide attempt or plan. Because Q13 was lifetime in timeframe, this domain was interpreted as a suicide-related screening signal rather than current suicidal behavior.

The psychotic-like symptom domain was defined as endorsement of at least one of Q14–Q16: hearing voices others cannot hear, feeling watched or that one’s thoughts are controlled without a clear reason, or believing in supernatural powers or abilities not possessed by others. These items were interpreted as psychotic-like screening signals requiring further assessment, not evidence of psychotic disorder. This cautious terminology is consistent with population research showing that psychotic experiences can occur outside psychotic disorders and may be shaped by culture, stress, and context [15,16,17].

The mood-instability/bipolar-spectrum domain was defined as endorsement of at least one of Q21–Q23: episodes of hyperactivity or excessive energy with reduced need for sleep, mood elevation or irritability noticed by others, or racing thoughts or unusually rapid speech. Q20, which assessed cognitive difficulty and distractibility, was excluded from this severe construct because it is nonspecific in Hajj field settings and may reflect fatigue, sleep deprivation, crowding, heat exposure, anxiety, or physical illness rather than mood instability.

A severe symptom cluster was defined as positivity in at least one of the three severe domains. This cluster was used as an operational screening construct to summarize crisis-relevant signals; it was not treated as a diagnosis or a validated severity scale. Covariates were sex, age group, marital status, current physical illness, and prior psychiatric history. Age 41–50 years was the reference group in adjusted models.

### 2.4. Statistical Analysis

Categorical variables were summarized as frequencies and percentages. Age was summarized using mean, standard deviation, median, interquartile range, and range. Prevalence estimates were calculated using the final analytic sample as the denominator, with two-sided Wilson 95% confidence intervals for proportions [18,19]. Domain overlap was described by counting the number of severe domains endorsed among cluster-positive participants.

Four separate multivariable logistic regression models estimated adjusted associations with suicide-related symptoms, psychotic-like symptoms, mood-instability/bipolar-spectrum symptoms, and the severe symptom cluster. All models included the same prespecified covariates. Adjusted odds ratios (AORs), 95% confidence intervals, and two-sided *p*-values were reported as effect-size estimates and measures of statistical uncertainty. AORs were interpreted as odds ratios rather than risk ratios. McFadden pseudo-R2 and area under the receiver operating characteristic curve (AUC) were used only as descriptive model summary measures to clarify model explanatory performance and discrimination; they were not used to derive prediction scores, risk strata, or triage rules [20,21]. Analyses were performed using Python 3.13.5 (Python Software Foundation, Wilmington, DE, USA), with pandas 2.2.3, statsmodels 0.14.6, and scikit-learn 1.8.0.

### 2.5. Ethical Considerations

The parent study was reviewed and approved by the Riyadh Second Health Cluster Institutional Review Board, King Fahad Medical City, Riyadh, Saudi Arabia (IRB Log Number: 25-256E; approval date: 12 May 2025; category: expedited). Written informed consent was obtained from all participants in accordance with IRB-approved procedures. The present manuscript reports a focused analysis of de-identified coded screening data and includes no identifiable individual-level information, images, or case descriptions.

## 3. Results

### 3.1. Participant Characteristics

The final analytic sample included 2171 adult pilgrims aged 18–95 years. The mean age was 49.5 years (SD 12.6), and the median age was 50.0 years (IQR 40.0–60.0). Men represented 54.7% of the sample, 89.2% were married, 23.6% reported current physical illness, and 2.8% reported prior psychiatric history (Table 1). The most frequent survey administration language/support streams were Arabic (39.2%), Indonesian (11.7%), and English (9.8%); other language-specific or interpreter-supported streams together accounted for 854 participants (39.3%).

### 3.2. Severe Screen-Positive Symptom Signals

**Figure 1:**
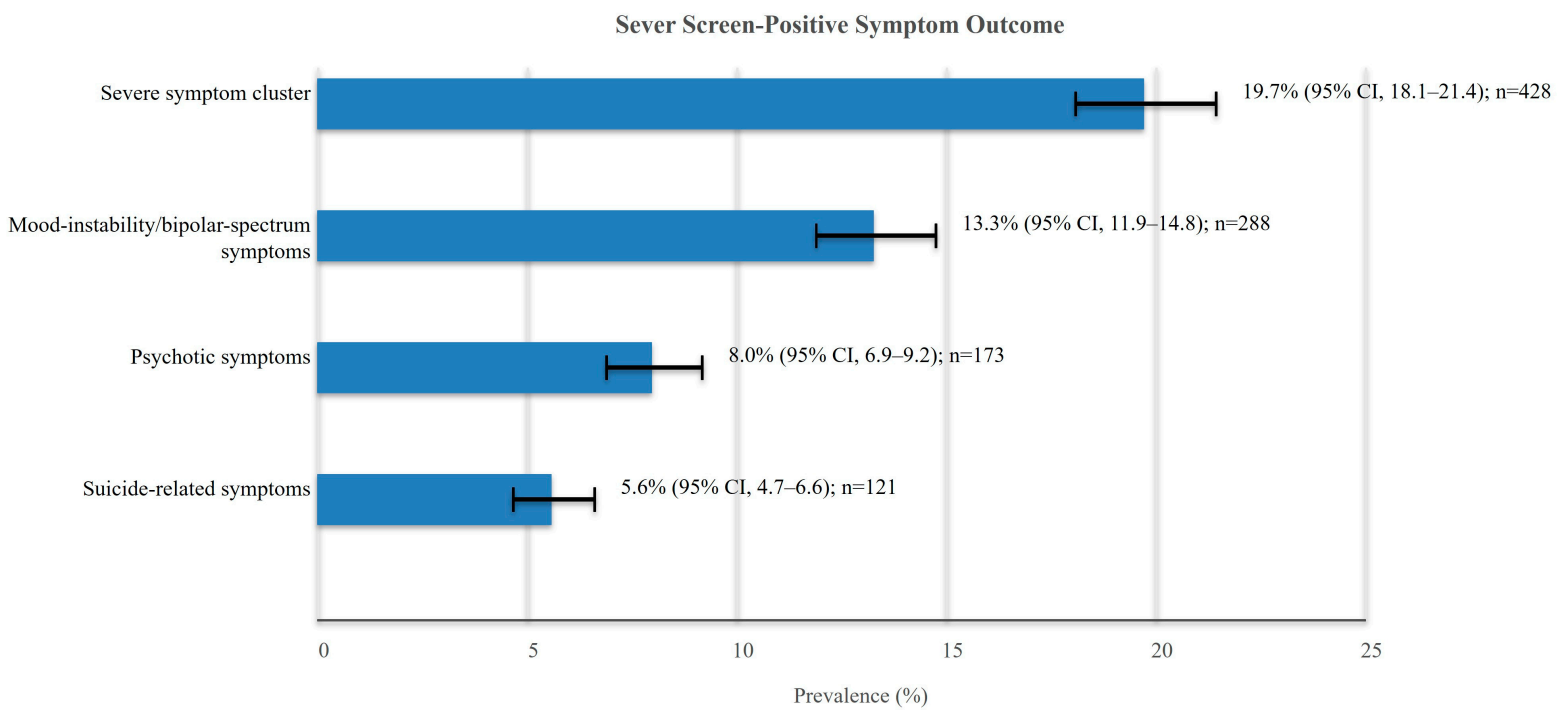
Prevalence of severe screen-positive symptom outcomes among adult Hajj pilgrims. Bars represent prevalence estimates and error bars represent Wilson 95% confidence intervals. Outcomes are screening signals rather than psychiatric diagnoses.

Severe screen-positive symptom signals were identified in 428 participants, corresponding to a severe symptom cluster prevalence of 19.7% (95% CI 18.1–21.4). Mood-instability/bipolar-spectrum symptoms were the most common severe-domain signal (13.3%; 95% CI 11.9–14.8), followed by psychotic-like symptoms (8.0%; 95% CI 6.9–9.2) and suicide-related symptoms (5.6%; 95% CI 4.7–6.6) (Table 2; Figure 1).

Within the suicide-related domain, thoughts about death or wishing to die during the past month were endorsed by 105 participants (4.8%), thoughts of self-harm or suicide by 22 participants (1.0%), and previous suicide attempt or plan by 18 participants (0.8%). Among psychotic-like items, belief in supernatural powers or abilities was most frequent (n = 129; 5.9%), followed by feeling watched or that thoughts were controlled (n = 48; 2.2%) and hearing voices others could not hear (n = 34; 1.6%). Among mood-instability/bipolar-spectrum items, the most frequent endorsement was hyperactivity or excessive energy with reduced need for sleep (n = 167; 7.7%). These item-level findings require cautious interpretation because item endorsement may be influenced by language, idiom, cultural interpretation, and interview context.

### 3.3. Domain Overlap

Among the 428 cluster-positive participants, 297 endorsed exactly one severe domain (69.4%), 108 endorsed two domains (25.2%), and 23 endorsed all three severe domains (5.4%). Thus, 131 cluster-positive participants (30.6%) endorsed two or more severe domains. Mood-instability/bipolar-spectrum symptoms only was the most frequent single-domain pattern. Mood instability with psychotic-like symptoms was the most frequent two-domain pattern (Table 3). Overlap was interpreted as co-endorsement of screening domains, not as a validated indicator of clinical severity; however, multi-domain endorsement may indicate greater referral complexity and the need for more coordinated safety review and handover.

### 3.4. Adjusted Associations

**Figure 2:**
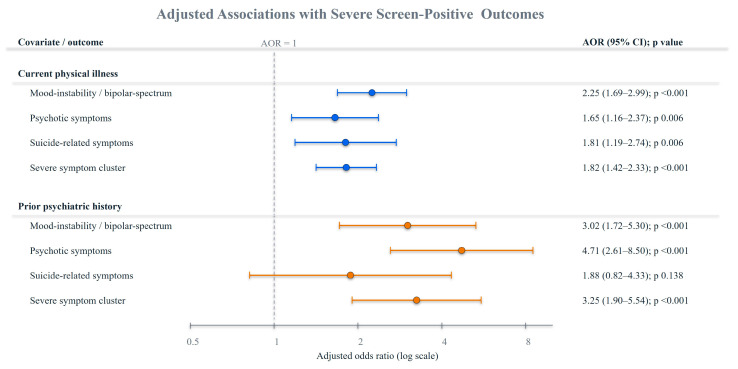
Adjusted associations of current physical illness and prior psychiatric history with severe screen-positive symptom outcomes. Points represent adjusted odds ratios and horizontal lines represent 95% confidence intervals from separate multivariable logistic regression models. Blue markers indicate current physical illness, and orange markers indicate prior psychiatric history. Models were not developed as clinical prediction tools.

Current physical illness was independently associated with all four severe outcomes: mood-instability/bipolar-spectrum symptoms (AOR 2.25; 95% CI 1.69–2.99), psychotic-like symptoms (AOR 1.65; 95% CI 1.16–2.37), suicide-related symptoms (AOR 1.81; 95% CI 1.19–2.74), and the severe symptom cluster (AOR 1.82; 95% CI 1.42–2.33). Prior psychiatric history was associated with mood-instability/bipolar-spectrum symptoms (AOR 3.02; 95% CI 1.72–5.30), psychotic-like symptoms (AOR 4.71; 95% CI 2.61–8.50), and the severe symptom cluster (AOR 3.25; 95% CI 1.90–5.54), but not independently with suicide-related symptoms (AOR 1.88; 95% CI 0.82–4.33; *p* = 0.138) (Table 4; Figure 2). In the full supplementary models, the age > 70 years group showed a non-significant directional association with lower odds of mood-instability/bipolar-spectrum symptoms compared with ages 41–50 years (AOR 0.48; 95% CI 0.21–1.11; *p* = 0.087). McFadden pseudo-R2 values were low (0.019–0.043) and model discrimination was modest (AUC range 0.610–0.640), so the models were interpreted as association models with limited explanatory and predictive performance.

## 4. Discussion

This study identified severe screen-positive mental health symptom signals in a clinically relevant minority of adult multinational Hajj pilgrims. Approximately one in five participants endorsed at least one severe domain, with mood-instability/bipolar-spectrum signals being the most frequent. These findings should not be interpreted as diagnostic prevalence. Their significance is operational and healthcare-related: in a multilingual mass-gathering environment, brief confidential interviews can identify signals that may otherwise remain hidden behind stigma, language barriers, physical complaints, or limited access to specialist mental healthcare.

The findings complement earlier Hajj psychiatric evidence rather than directly replicating it. The MINI-based study by Alzahrani et al. [13] estimated psychiatric disorders and suicide risk using a diagnostic instrument in a smaller sample. The present study used brief field screening items, a larger multinational sample, and an operational severe-cluster definition. The contribution is therefore not that the study diagnoses psychiatric disorders during Hajj; it shows that crisis-relevant screening signals can be detected during brief multilingual field encounters and can inform service planning and clinical vigilance.

The overlap findings also have operational significance. Participants endorsing two or three severe domains may require more than a single screening response: for example, suicide-related items may trigger safety review, psychotic-like items may require culturally sensitive clarification and clinical assessment, and mood-instability items may require evaluation of sleep loss, exhaustion, behavioral activation, or medical contributors. In a high-volume multilingual Hajj setting, this co-occurrence can increase the demand for interpreter-supported assessment, field coordination, documentation, and referral prioritization. Therefore, multi-domain endorsement should be treated as a service-planning and handover signal, while remaining distinct from a diagnostic severity scale.

The association between current physical illness and all severe outcomes is particularly important from a healthcare-access perspective. In Hajj, pilgrims with physical illness may interact with health services more frequently than those with primarily psychological distress. Physical health encounters may therefore create opportunities for sensitive mental health inquiry, especially when distress is somatized, concealed, normalized, or difficult to communicate across languages. The finding is compatible with broader evidence linking physical illness and mental health burden [22,23], but its field implication is specific: general medical and emergency encounters during mass gatherings should be designed to accommodate discreet mental health screening and referral. This interpretation is consistent with recent Hajj healthcare evidence in which field-level flags were associated with care intensity and referral documentation, but were explicitly framed as service-planning prompts rather than validated individual-level prediction tools [6].

The absence of an independent association between prior psychiatric history and suicide-related symptoms after adjustment also has practical implications. If field teams rely mainly on known psychiatric history, they may miss individuals endorsing suicide-related screening items. This does not mean that psychiatric history is unimportant; it was strongly associated with psychotic-like and mood-instability signals. Rather, it suggests that safety-oriented questioning should be available when distress, severe screening responses, physical illness burden, or observed concern emerges, even in the absence of a disclosed psychiatric history. Such questioning must be confidential, culturally sensitive, and linked to referral pathways; screening tools should inform, not replace, clinical judgment [11,24].

The psychotic-like and mood-instability findings require careful interpretation. Psychotic-like experiences occur in the general population and are not synonymous with psychotic disorder [16,17]. In pilgrimage settings, culturally shaped spiritual language, stress, sleep disruption, fatigue, interpersonal concerns, and translation effects may influence responses. Similarly, endorsement of hyperactivity, mood elevation, racing thoughts, or reduced need for sleep does not establish mania or bipolar disorder. The high endorsement of hyperactivity or excessive energy with reduced sleep may partly reflect the physical exertion, disrupted sleep, prolonged walking, heat exposure, and intensive ritual schedule of Hajj rather than a primary bipolar-spectrum condition. The non-significant lower odds of mood-instability endorsement among participants aged >70 years may reflect sample composition, selective participation, survivorship, or age-related differences in symptom disclosure, and should be interpreted cautiously. These domains are best understood as prompts for further review when clinically indicated, not as diagnostic labels.

The study also highlights the importance of interpreter-supported and culturally aware screening. A multilingual survey alone is insufficient if interviewers cannot manage ambiguous expressions, distress, refusal, embarrassment, or culturally loaded terms. Separating the roles of tool reviewers and field translators, using brief interviews in semi-private settings, and providing stop-and-refer instructions for severe disclosures were practical safeguards. Future mass-gathering healthcare work should evaluate whether such procedures improve referral reliability, patient acceptability, and continuity of care after pilgrims return to their home countries.

Several limitations should be considered. The study was cross-sectional and cannot infer causality. Outcomes were based on screening items rather than diagnostic interviews, and no clinical verification of psychiatric diagnoses was available. Suicide-related items included different timeframes, including a lifetime attempt or plan item, so this domain should not be interpreted as current suicidal behavior. Sampling used a pragmatic accommodation-site field approach rather than probability sampling, so selection bias is possible and the sample should not be treated as representative of all Hajj pilgrims. Current physical illness and prior psychiatric history were self-reported and not clinically verified. Disclosure bias, stigma, language differences, grouped reporting of less frequent language/support streams, and interviewer effects may have influenced responses. Finally, the low pseudo-R2 values and modest AUC values indicate that the regression models should not be used as prediction tools or triage scores.

Despite these limitations, the study has strengths relevant to healthcare delivery during mass gatherings: a large multinational field sample, interview administration rather than self-completion, multilingual procedures, prespecified severe domains, clear separation between screening and diagnosis, and adjusted analyses using the same covariates across outcomes. The findings support integrating confidential severe symptom inquiry into general Hajj healthcare encounters, with multilingual communication and referral pathways available when screening signals raise concern.

## 5. Conclusions

Severe screen-positive mental health symptom signals were present in a clinically meaningful minority of adult multinational Hajj pilgrims. Current physical illness was the most consistent correlate across severe outcomes, while prior psychiatric history did not independently identify suicide-related symptoms. These findings support confidential, culturally sensitive, and multilingual severe symptom screening within Hajj healthcare encounters, especially where physical illness brings pilgrims into contact with services. Screen-positive findings require further clinical assessment and should not be interpreted as psychiatric diagnoses.

## Figures and Tables

**Table 1:** Participant characteristics and survey administration profile.

Characteristic	n (%) or Summary
Age, years, mean ± SD	49.5 ± 12.6
Median (IQR)	50.0 (40.0–60.0)
Range	18–95
Age 18–30 years	163 (7.5)
Age 31–40 years	415 (19.1)
Age 41–50 years	558 (25.7)
Age 51–60 years	608 (28.0)
Age 61–70 years	348 (16.0)
Age > 70 years	79 (3.6)
Male sex	1187 (54.7)
Female sex	984 (45.3)
Married	1937 (89.2)
Unmarried	234 (10.8)
Current physical illness	513 (23.6)
Prior psychiatric history	60 (2.8)
Arabic administration/support stream	850 (39.2)
Indonesian administration/support stream	255 (11.7)
English administration/support stream	212 (9.8)
Other language/support streams, combined	854 (39.3)

Note. Values are n (%) unless otherwise indicated. The analytic sample included 2171 adult pilgrims after exclusion of three participants younger than 18 years. Language/support streams are field-administration categories; less frequent language-specific or interpreter-supported streams are grouped as Other.

**Table 2:** Severe screen-positive symptom prevalence by domain and selected item-level findings.

Outcome or Item	Definition	n	%	95% CI or Note
Severe symptom cluster	Positive in ≥1 severe domain	428	19.7	18.1–21.4
Mood-instability/bipolar-spectrum domain	Positive in Q21–Q23	288	13.3	11.9–14.8
Psychotic-like symptom domain	Positive in Q14–Q16	173	8.0	6.9–9.2
Suicide-related symptom domain	Positive in Q11–Q13	121	5.6	4.7–6.6
Thoughts about death/wishing to die	Q11 item endorsement	105	4.8	Item-level screening signal
Thoughts of self-harm or suicide	Q12 item endorsement	22	1.0	Item-level screening signal
Previous suicide attempt or plan	Q13 item endorsement	18	0.8	Lifetime item
Hearing voices others cannot hear	Q14 item endorsement	34	1.6	Psychotic-like signal
Feeling watched/thoughts controlled	Q15 item endorsement	48	2.2	Psychotic-like signal
Belief in supernatural powers/abilities	Q16 item endorsement	129	5.9	Culturally sensitive interpretation needed
Hyperactivity/excessive energy with reduced sleep	Q21 item endorsement	167	7.7	Mood-instability signal

Note. Percentages use n = 2171 as denominator unless otherwise stated. Outcomes are screen-positive symptom signals, not psychiatric diagnoses.

**Table 3:** Domain overlap among severe-cluster-positive participants.

Overlap Pattern	Definition	n	% of Cluster-Positive
Exactly one severe domain	Among severe-cluster-positive participants	297	69.4
Any two severe domains	Among severe-cluster-positive participants	108	25.2
All three severe domains	Among severe-cluster-positive participants	23	5.4

Note. Cluster-positive n = 428. Domain overlap is not a validated severity scale.

**Table 4:** Key adjusted associations with severe screen-positive symptom outcomes.

Covariate	Mood Instability	Psychotic-Like Symptoms	Suicide-Related Symptoms	Severe Cluster
Current physical illness	2.25 (1.69–2.99)	1.65 (1.16–2.37)	1.81 (1.19–2.74)	1.82 (1.42–2.33)
Prior psychiatric history	3.02 (1.72–5.30)	4.71 (2.61–8.50)	1.88 (0.82–4.33)	3.25 (1.90–5.54)

Note. Values are AORs (95% CI). Models adjusted for sex, age group, marital status, current physical illness, and prior psychiatric history. Age 41–50 years was the reference group. Full model results are provided in Supplementary Table S3.

## Data Availability

The datasets analyzed during the current study are not publicly available because they contain sensitive mental health screening data collected during Hajj and are subject to institutional and ethics restrictions. De-identified data may be considered only upon reasonable request to the corresponding author, subject to approval by the relevant ethics committee and institutional data governance requirements.

## Supplementary Materials

The following supporting information can be downloaded at https://www.mdpi.com/article/10.3390/healthcare14152276/s1, Table S1: Severe symptom screening item domains; Table S2: Model summary statistics; Table S3: Full adjusted logistic regression models.

## References

[1] World Health Organization (2015). Public Health for Mass Gatherings: Key Considerations.

[2] Memish Z.A., Stephens G.M., Steffen R., Ahmed Q.A. (2012). Emergence of medicine for mass gatherings: Lessons from the Hajj. Lancet Infect. Dis..

[3] Aldossari M., Aljoudi A., Celentano D. (2019). Health issues in the Hajj pilgrimage: A literature review. East. Mediterr. Health J..

[4] Rahman J., Thu M., Arshad N., van der Putten M. (2017). Mass gatherings and public health: Case studies from the Hajj to Mecca. Ann. Glob. Health.

[5] Steffen R., Bouchama A., Johansson A., Dvorak J., Isla N., Smallwood C., Memish Z.A. (2012). Non-communicable health risks during mass gatherings. Lancet Infect. Dis..

[6] AlGabgab M.F., Alqurashi N., Alqahtani M., Alolyani M.M., Samarkandi O.A. (2026). Core High-Risk Foot Profiles and Surgery-Coded Care-Intensity Indicators Among Hajj Pilgrims Presenting with Foot and Ankle Conditions: A Presentation-Level Analysis. Healthcare.

[7] Khan S.A., Chauhan V.S., Timothy A., Kalpana S., Khanam S. (2016). Mental health in mass gatherings. Ind. Psychiatry J..

[8] Hankir A., Chariwala Z., Siddique U., Carrick F.R., Zaman R. (2019). Hajj and the mental health of Muslim pilgrims: A review. Psychiatr. Danub..

[9] World Health Organization, War Trauma Foundation, World Vision International (2011). Psychological First Aid: Guide for Field Workers.

[10] World Health Organization (2016). mhGAP Intervention Guide for Mental, Neurological and Substance Use Disorders in Non-Specialized Health Settings.

[11] Ministry of Health, Kingdom of Saudi Arabia (2023). Protocol for Suicide Risk Assessment and Management in MOH Facilities.

[12] American Psychiatric Association (2013). Diagnostic and Statistical Manual of Mental Disorders.

[13] Alzahrani A.S., Alqahtani A.M., Elmorsy S.A., Alhazmi M., Mahdi H.A., Albarakati B., Alkhiri A., Hakeem A. (2021). Prevalence of psychiatric disorders in Hajj pilgrims using MINI as a diagnostic tool. J. Public Health.

[14] Masood K., Gazzaz Z.J., Ismail K., Dhafar K.O., Kamal A. (2007). Pattern of psychiatry morbidity during Hajj period at Al-Noor Specialist Hospital. Int. J. Psychiatry Med..

[15] Kirmayer L.J., Pedersen D. (2014). Toward a new architecture for global mental health. Transcult. Psychiatry.

[16] McGrath J.J., Saha S., Al-Hamzawi A., Andrade L., Benjet C., Bromet E.J., Bruffaerts R., Caldas-De-Almeida J.M., Chiu W.T., De Jonge P. (2015). Psychotic experiences in the general population: A cross-national analysis based on 31,261 respondents from 18 countries. JAMA Psychiatry.

[17] Staines L., Healy C., Coughlan H., Clarke M., Kelleher I., Cotter D., Cannon M. (2022). Psychotic experiences in the general population: A review; definition, risk factors, outcomes and interventions. Psychol. Med..

[18] Newcombe R.G. (1998). Two-sided confidence intervals for the single proportion: Comparison of seven methods. Stat. Med..

[19] Wilson E.B. (1927). Probable inference, the law of succession, and statistical inference. J. Am. Stat. Assoc..

[20] McFadden D., Zarembka P. (1974). Conditional logit analysis of qualitative choice behavior. Frontiers in Econometrics.

[21] Steyerberg E.W. (2019). Clinical Prediction Models: A Practical Approach to Development, Validation, and Updating.

[22] Moussavi S., Chatterji S., Verdes E., Tandon A., Patel V., Ustun B. (2007). Depression, chronic diseases, and decrements in health: Results from the World Health Surveys. Lancet.

[23] Guo S., Qing G., Yang G. (2024). The relationship between chronic disease variety and quantity and suicidal ideation: A cross-sectional study of NHANES. J. Psychosom. Res..

[24] US Preventive Services Task Force (2023). Screening for depression and suicide risk in adults: US Preventive Services Task Force recommendation statement. JAMA.

