# Supplementary material for: Severe Mental Health Screening Signals Among Multinational Hajj Pilgrims in 2025: Implications for Multilingual Healthcare Encounters and Referral Planning
Source: Healthcare (Basel). 2026 Jul 25;14(15):2276. doi: 10.3390/healthcare14152276 (PMC13463963; doi:10.3390/healthcare14152276)
Supplement: Supplementary file 1 [file healthcare-14-02276-s001.zip › healthcare-4422878-supplementary.pdf]

Supplementary Material

Severe Mental Health Screening Signals Among Multinational Hajj Pilgrims in 2025: Implications for Multilingual Healthcare Encounters and Referral Planning

Supplementary Table S1. Severe symptom screening item domains.

Domain	Items	Operational definition
Suicide-related symptoms	Q11-Q13	Endorsement of at least one suicide-related screening item.
Psychotic-like symptoms	Q14-Q16	Endorsement of at least one psychotic-like screening item.
Mood-instability/bipolar-spectrum symptoms	Q21-Q23	Endorsement of at least one mood-instability/bipolar-spectrum screening item.
Severe symptom cluster	Any of the above	Endorsement of at least one item in any of the three severe domains.

Supplementary Table S2. Model summary statistics.

Outcome	Events, n	McFadden pseudo-R2	AUC	Interpretation
Mood-instability/bipolar-spectrum symptoms	288	0.043	0.640	Association model; not a prediction tool
Psychotic-like symptoms	173	0.036	0.622	Association model; not a prediction tool
Suicide-related symptoms	121	0.019	0.610	Association model; not a prediction tool
Severe symptom cluster	428	0.032	0.610	Association model; not a prediction tool

Supplementary Table S3. Full adjusted logistic regression models for severe screen-positive outcomes.

Covariate	Reference	Mood-instability	Psychotic-like	Suicide-related	Severe cluster
Female sex	Male	1.44 (1.11-1.86); p=0.006	1.12 (0.82-1.55); p=0.470	1.05 (0.72-1.53); p=0.784	1.17 (0.94-1.46); p=0.153
Unmarried status	Married	1.45 (1.01-2.09); p=0.047	1.34 (0.84-2.14); p=0.212	1.35 (0.79-2.29); p=0.268	1.68 (1.22-2.30); p=0.001
Current physical illness	No current physical illness	2.25 (1.69-2.99); p<0.001	1.65 (1.16-2.37); p=0.006	1.81 (1.19-2.74); p=0.006	1.82 (1.42-2.33); p<0.001
Prior psychiatric history	No prior psychiatric history	3.02 (1.72-5.30); p<0.001	4.71 (2.61-8.50); p<0.001	1.88 (0.82-4.33); p=0.138	3.25 (1.90-5.54); p<0.001
Age 18-30 years	Age 41-50 years	1.48 (0.90-2.45); p=0.125	1.13 (0.60-2.12); p=0.701	2.08 (1.00-4.30); p=0.049	1.42 (0.91-2.19); p=0.119
Age 31-40 years	Age 41-50 years	1.22 (0.84-1.78); p=0.302	0.81 (0.50-1.31); p=0.383	1.62 (0.91-2.89); p=0.104	1.17 (0.84-1.63); p=0.344
Age 51-60 years	Age 41-50 years	0.86 (0.60-1.23); p=0.402	0.81 (0.53-1.24); p=0.335	1.41 (0.82-2.42); p=0.209	1.05 (0.78-1.42); p=0.740
Age 61-70 years	Age 41-50 years	0.83 (0.55-1.26); p=0.387	0.69 (0.40-1.16); p=0.162	1.05 (0.55-2.02); p=0.884	0.97 (0.68-1.39); p=0.880

Covariate	Reference	Mood-instability	Psychotic-like	Suicide-related	Severe cluster
Age >70 years	Age 41-50 years	0.48 (0.21-1.11); p=0.087	1.25 (0.59-2.65); p=0.562	1.02 (0.34-3.06); p=0.972	0.71 (0.37-1.36); p=0.300

Note. Values are adjusted odds ratios with 95% confidence intervals and two-sided p-values. Four separate multivariable logistic regression models were fitted. Outcomes represent screen-positive symptom signals rather than psychiatric diagnoses. The age >70 years term in the mood-instability model (AOR 0.48; 95% CI 0.21-1.11; p=0.087) was interpreted as a non-significant directional pattern and not as evidence of a statistically significant protective effect.
